# Jinfukang inhibits clustering and invasion of circulating lung tumor cells by regulating the EGFR signaling pathway: Anti-metastatic mechanism of Jinfukang on lung cancer

**DOI:** 10.3724/abbs.2023148

**Published:** 2023-09-18

**Authors:** Yun Yang, Lihua Zhu, Jiajun Liu, Pan Yu, Zujun Que, Yan Li, Hegen Li, Jianhui Tian

**Affiliations:** 1 Clinical Oncology Center Shanghai Municipal Hospital of Traditional Chinese Medicine Shanghai University of Traditional Chinese Medicine Shanghai 200071 China; 2 Department of Oncology Longhua Hospital Shanghai University of Traditional Chinese Medicine Shanghai 200032 China; 3 Institute of Oncology Shanghai Municipal Hospital of Traditional Chinese Medicine Shanghai University of Traditional Chinese Medicine Shanghai 200071 China

Lung cancer is the leading cause of cancer-related death in China and worldwide [1]. Although the popularization and application of PET-CT have improved the detection rate of patients with early lung cancer, there is still a lack of effective drugs to prevent and treat lung cancer metastasis and prolong the survival of patients [ 2, 3] . Circulating tumor cells (CTCs), as seeds of tumor metastasis, play an important role in the metastasis of lung cancer. Many studies have demonstrated that CTC level is correlated with distant metastasis and poor prognosis in lung cancer [4]. In addition, an increase in the number of CTCs during treatment in patients with early-stage lung cancer after surgery indicates that the disease has progressed [5]. Aceto *et al*. [6] found that the expression of plakoglobin (JUP) was significantly upregulated on CTC clusters, and at the same time, the metastatic potential of CTC clusters was increased by 23- to 50-fold compared with that of single CTCs. JUP is a member of the Armadillo family of proteins and a key component of intercellular junctions. Knockdown of *JUP* abrogated the formation of breast cancer CTC clusters and suppressed lung metastases [6]. Therefore, inhibiting CTC clustering may be a treatment strategy against lung cancer metastasis.

Traditional Chinese medicine has been used in China for thousands of years. It is used to treat various diseases in the clinic. Jinfukang is a Chinese herbal prescription for the treatment of lung cancer that was approved by the State Food and Drug Administration in 1996 (Z19991043). Multiple lines of evidence indicated that Jinfukang can prevent metastasis and prolong the survival of lung cancer patients in the clinic [7]. In addition, Jinfukang can induce cell cycle arrest, apoptosis, and epigenetic regulation in lung cancer cells [8]. Our laboratory and Key Laboratory of Systems Biomedicine at Shanghai Jiao Tong University have established the human circulating lung tumor cell line CTC-TJH-01 to explore the inhibitory effect and mechanism of Jinfukang on CTCs [ 9, 10] . Jinfukang was found to induce anoikis in CTCs, thereby inhibiting their metastasis [11]. However, whether Jinfukang can regulate the clustering of CTCs in peripheral blood is still unknown. In this study, we examined the effect of Jinfukang on CTC clustering and revealed its regulatory mechanism.

**Figure 1:**
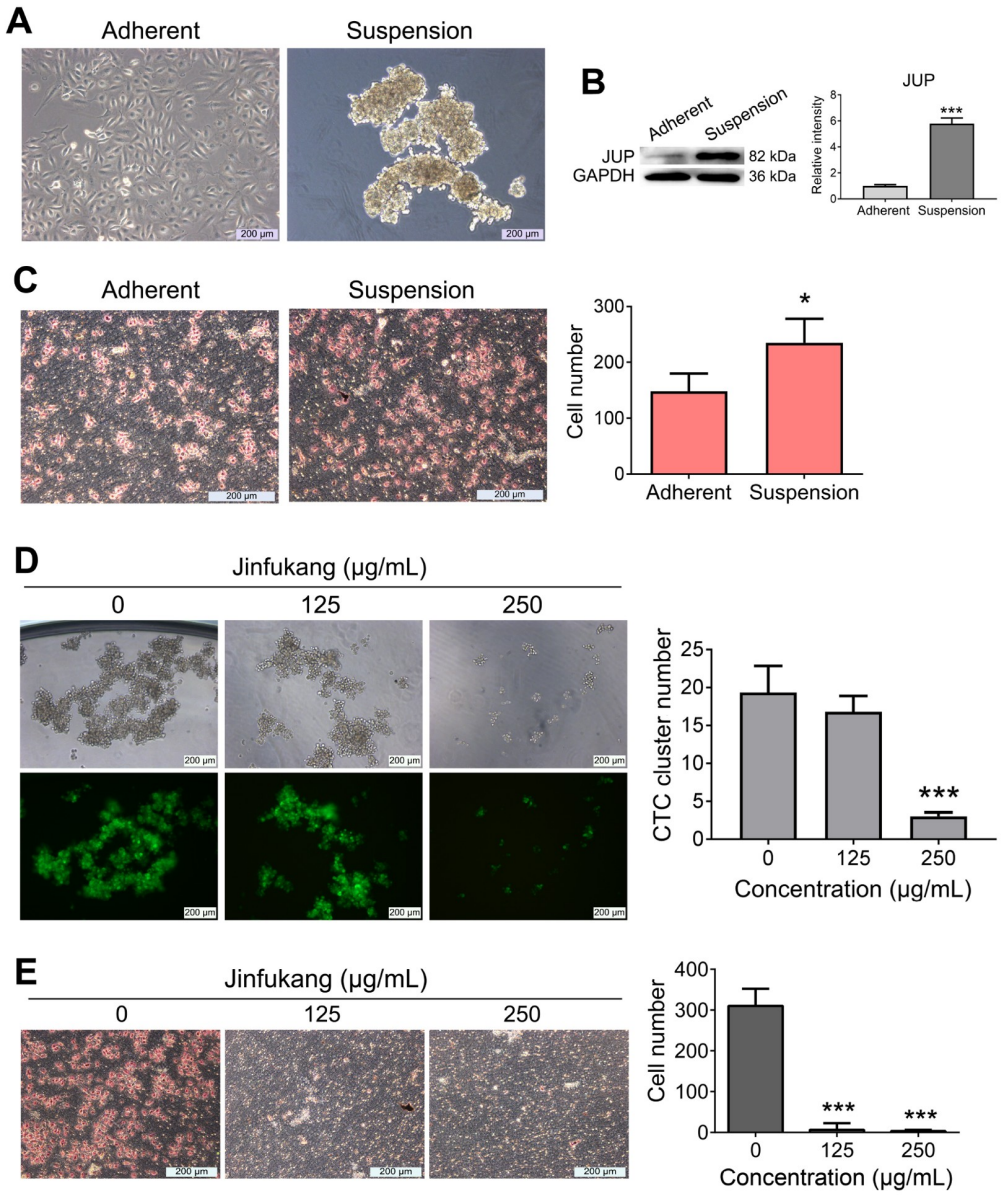
Figure 1 Effects of Jinfukang on CTC clustering and invasion (A) Morphological observation of CTC-TJH-01 cells in adherent and suspension cultures. (B) Western blot analysis was used to detect the expression of JUP protein in adherent and suspended CTC-TJH-01 cells. GAPDH was usde as a control. (C) Comparison of migration ability between adherent and suspension-cultured CTC-TJH-01 cells. (D) CTC-TJH-01 cells cultured in suspension were treated with different concentrations of Jinfukang (0, 125, and 250 μg/mL) for 48 h. CTC-TJH-01 cell clusters were observed and counted under an inverted fluorescence microscope. (E) Cell invasion assay was used to detect the invasion ability of CTC-TJH-01 cell clusters after treatment with Jinfukang. Data are presented as the mean±SD of three independent experiments. * P<0.05, *** P<0.001.

CTC-TJH-01 cells were seeded in 6-well plates treated with poly-HEMA using complete medium for 48 h to build a suspension culture system. Single CTC-TJH-01 cells aggregated to form clusters in suspension culture ( Figure 1A). Compared with CTC-TJH-01 cells in adherent culture, the expression of JUP protein was significantly increased in suspension-cultured CTC-TJH-01 cell clusters ( Figure 1B). In addition, our results indicated that the invasion potential was enhanced ( Figure 1C). Whether Jinfukang plays an antimetastatic role in lung cancer by regulating CTC clustering is still unknown. Our study revealed that high concentration of Jinfukang significantly inhibited the clustering ability of CTC-TJH-01-GFP cells, showing that the diameter of cell clusters was significantly reduced, and the compactness between cells was also reduced ( Figure 1D). In addition, both high and low concentrations of Jinfukang significantly inhibited the invasion of CTC-TJH-01-GFP cell clusters ( Figure 1E). These results suggest that CTC clusters overexpress JUP protein and have enhanced invasion potential in lung cancer, while Jinfukang can inhibit CTC clustering and invasion.

**Figure 2:**
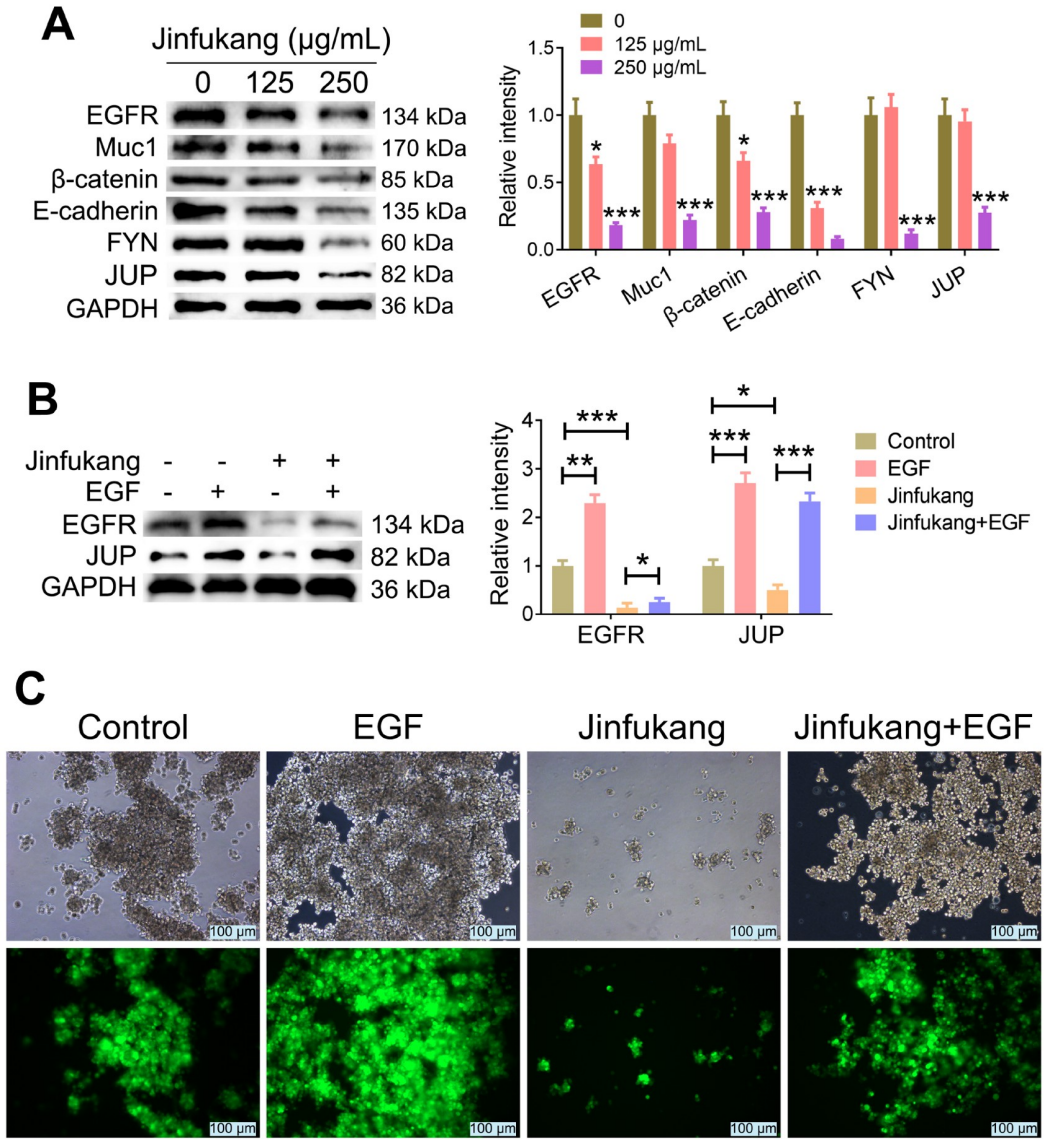
Figure 2 Effect of Jinfukang on the EGFR signaling pathway in CTC clusters (A) CTC-TJH-01 cells cultured in suspension were treated with different concentrations of Jinfukang (0, 125, and 250 μg/mL) for 48 h, and western blot analysis was performed to determine EGFR, Muc1, β-catenin, E-cadherin, FYN, and JUP protein expressions. GAPDH was used as a control. (B) The effect of EGF (10 μg/mL) and Jinfukang (250 μg/mL) single and combination treatments on the expressions of EGFR and JUP protein in CTC-TJH-01 cell clusters was determined by western blot analysis. GAPDH was used as a control. (C) The effect of EGF (10 μg/mL) and Jinfukang (250 μg/mL) single and combination treatments on CTC-TJH-01 cell clustering was observed by inverted fluorescence microscopy. Scale bar: 100 μm. Data are presented as the mean±SD of three independent experiments. * P<0.05, ** P<0.01, *** P<0.001.

To reveal the mechanism by which Jinfukang inhibits CTC clustering and invasion in lung cancer, we first detected JUP protein, which regulates the clustering of CTCs, and found that high concentration of Jinfukang significantly downregulated the expression of JUP protein in CTC-TJH-01-GFP cell clusters ( Figure 2A). Next, we analyzed the cytoskeletal regulatory pathways that can regulate the expression of the JUP protein. Jinfukang significantly downregulated the protein expressions of EGFR, Muc1, β-catenin, E-cadherin and FYN in CTC-TJH-01-GFP cell clusters ( Figure 2A). We next used EGF to activate the EGFR pathway to confirm the mechanism by which Jinfukang inhibits JUP protein expression and CTC-TJH-01-GFP cell clustering. We found that the expression of JUP protein was also significantly upregulated when the expression of EGFR on CTC-TJH-01 cell clusters was upregulated with EGF ( Figure 2B). In addition, when CTC-TJH-01 cell clusters were cotreated with EGF and Jinfukang, EGF reversed the inhibitory effect of Jinfukang on the protein expressions of EGFR and JUP ( Figure 2B). Furthermore, we also found that EGF promoted the clustering of CTC-TJH-01 cells and reversed the inhibitory effect of Jinfukang on CTC-TJH-01 cell clustering ( Figure 2C). These results proved that Jinfukang downregulates the expression of JUP by regulating the EGFR pathway and then inhibits CTC clustering and invasion in lung cancer.

In summary, a CTC suspension culture system was established by using poly-HEMA-treated 6-well plates to investigate the inhibitory effect and mechanism of Jinfukang on CTCs. Our study confirmed that Jinfukang has inhibitory effects on the clustering and invasion of lung cancer CTCs, mainly by regulating the EGFR-mediated cytoskeleton regulation pathway to downregulate the expression of JUP protein. The above findings provide a new mechanism for the effect of Jinfukang in the treatment of lung cancer metastasis.
